# An integration of hybrid MCDA framework to the statistical analysis of computer-based health monitoring applications

**DOI:** 10.3389/fpubh.2023.1341871

**Published:** 2024-01-08

**Authors:** Wang Hongxia, Guo Juanjuan, Wang Han, Lan Wenlong, Muhammad Yasir, Li Xiaojing

**Affiliations:** ^1^Qingdao Municipal Center for Disease Control and Prevention, Qingdao, China; ^2^Affiliated Qingdao Third People’s Hospital, Qingdao University, Qingdao, China; ^3^College of Oceanography and Space Informatics, China University of Petroleum, Qingdao, China

**Keywords:** additive ratio assessment, analytic hierarchy process, multi-criteria decision analysis, artificial intelligence, internet of things, health monitoring applications, computer technology and medical care

## Abstract

The surge in computer-based health surveillance applications, leveraging technologies like big data analytics, artificial intelligence, and the Internet of Things, aims to provide personalized and streamlined medical services. These applications encompass diverse functionalities, from portable health trackers to remote patient monitoring systems, covering aspects such as heart rate tracking, task monitoring, glucose level checking, medication reminders, and sleep pattern assessment. Despite the anticipated benefits, concerns about performance, security, and alignment with healthcare professionals’ needs arise with their widespread deployment. This study introduces a Hybrid Multi-Criteria Decision Analysis (MCDA) paradigm, combining the strengths of Additive Ratio Assessment (ARAS) and Analytic Hierarchy Process (AHP), to address the intricate nature of decision-making processes. The method involves selecting and structuring criteria hierarchically, providing a detailed evaluation of application efficacy. Professional stakeholders quantify the relative importance of each criterion through pairwise comparisons, generating criteria weights using AHP. The ARAS methodology then ranks applications based on their performance concerning the weighted criteria. This approach delivers a comprehensive assessment, considering factors like real-time capabilities, surgical services, and other crucial aspects. The research results provide valuable insights for healthcare practitioners, legislators, and technologists, aiding in deciding the adoption and integration of computer-based health monitoring applications, ultimately enhancing medical services and healthcare outcomes.

## 1 Introduction

The significant transformation the healthcare industry has undergone as a result of technological advancements is the direct cause of the development of computer-based health tracking software. These innovative applications offer a variety of benefits, including ongoing data collection, remote patient monitoring, and specialized medical services. Doctors and nurses are now better equipped to observe and operate patients’ health thanks to the smooth installation and usage of these apps with handheld gadgets, cell phones, and other sophisticated technologies. When these revolutionary resolves are put into practice, both patients and healthcare professionals feel strengthened since they have the ability to utilize crucial observations and resources for effectively managing, enforcing, and improving the quality of life. Computerized health monitoring systems are becoming increasingly prevalent, which is due to all of their beneficial features. These tools give individuals the comfort, mobility, and chance to take an active role in managing their particular health. Patients may anticipate health issues and make sound decisions about their condition by receiving constantly updated inputs and relevant responses. For healthcare professionals, these applications offer major data points that help with the creation of more customized and intelligent therapeutic activities. Doctors and nurses may give immediate assistance and distant therapy by using real-time patient health measurements and patterns. This improves patient satisfaction and relieves pressure on conventional medical structures. It is becoming more and more clear that computer-based health monitoring systems have the potential to change how healthcare is delivered as their use grows more prevalent. However, an extensive assessment of these apps’ efficacy, ease of use, and meeting marketplace requirements is essential given their increasing growth. It is crucial to take a scientific and evidence-based strategy to assess the performance of these apps given the variety of applications that are now accessible, each with its own distinct characteristics. Intending to deliver helpful knowledge for healthcare practitioners and stakeholders about well-informed decisions, our study attempts to address the major concern of evaluating and scrutinizing these applications. This study aims to determine the benefits and downsides of various apps, determine whether they are suited for certain healthcare situations using MCDA approaches, and offer improvements for future advancement through a thorough analysis.

The investigation that Sundström et al. (1) have proposed seeks to offer a survey of understanding and expertise gaps in the area of computer-based drugs measures by collecting data on the clinical efficacy of computer-based alcoholic beverages measures in various demographics and analyzing the implications of four specific facilitators of performance, medicinal direction, length of the treatment, assistance, and trial involvement. A collection of computer-based alcoholic beverage remedies investigated in thorough analyses of controlled studies between 2005 and 2015. Computer-based alcohol treatments have been viewed as useful in lowering alcohol intake in all of the relevant assessments, with the majority of the effect strengths being limited. Longer, multisession therapies may be more beneficial than less lengthy one-session ones, according to a previous study. The suggested thorough assessment identifies several kinds of topical gaps that may help direct future research in this area while also highlighting the overwhelmingly favorable research in favor of computer-based alcohol rehabilitation. The widespread adoption of wearable technology and bio-signal instruments, as well as the growth of the IoT and networking technologies, have all contributed to substantial advancements in the field of the quantified self. A number of platforms and services are using the premise, even if they are all primarily targeted toward certain industries like everyday usage and professions like athletics and healthcare. The proposed research (2) takes advantage of the AGILE IoT gateway’s open design, flexibility, and extension abilities to offer a multifaceted, cross-domain platform for creating services for the quantified self.

Effective medical services are needed to sustain individual health due to older people and the rise in chronic diseases globally. The suggested study offers a way for converting data collected by sensors into real-time therapeutic input by analyzing IoT-based medical services. This strategy takes a number of factors into account, namely mining, transmitting, analyzing, preserving, and detecting. By choosing to embrace a strategy that enables the creation of systems that are both practical and efficient, the developers will be able to go forward with system design for IoT-based medical purposes. The review’s findings indicate that predictive modeling, data mining, and personal care are the three IoT uses for medicine that are expanding the fastest (3). A novel strategy in the field of medicine is accessible healthcare. The accessible intelligent hospital, which includes a health screening system, and the accessible intelligent house are two of the most intriguing uses for accessible healthcare. Pharmaceutical staff members are kept up to date on the most recent computer-based advancements for enhancing patient care and streamlining hospitals thanks to medical technology. Clinics and other intelligent residences are being distributed around the country via an innovative, news-style strategy. Kemis et al. (4) perform this by developing a medical screening platform for widely scattered sensor systems. The pulse detector connects to the web server and transmits data there using the wireless RN-XV device, which depends on 802.11 standards. The health information that has been gathered may be accessed and reviewed by a doctor or nurse from a distance.

The severe biological disorder diabetes mellitus, also known as diabetes, currently affects people all around the world. This is crucial for a strong framework that can more correctly and quickly anticipate diabetes and its changes. Based on the size and complexity of the present body of research, hospitals urgently require appropriate and rapid disease assessments. Considering the present situation, it is imperative to develop mechanisms that are more adaptable, precise, trustworthy, and responsive. Sharma et al. (5) propose a ground-breaking approach to forecast accuracy using machine learning and AI. The suggested framework classifies diabetes according to the indicators in the data set, where each row represents a system rule that has to be comprehended and assembled using an attribute. In this regard, this study is an essential and pertinent attempt within the setting of cutting-edge healthcare. This study intends to advance health technology and encourage the incorporation of cutting-edge solutions into conventional medical practices through a thorough examination and analysis of the potential of such applications. The long-term objective is to strengthen the satisfaction of patients, encourage people to take charge of their health, and define the next generation of medical services through technological innovations. The existing literature contain the issues such as lack of integrated MCDA models, unspecified assessment factors, need for comparative significance analysis, lack of comprehensive prioritization methods, limited validation of assessment models, insufficient guidance for healthcare experts, limited advancement in assessment processes, and so on. The proposed research tackle the issues and provide the following contributions:

- To develop and implement an integrated MCDA model that combines the strengths of the AHP and ARAS approaches. The suggested framework would provide an organized and scientific process to evaluate and rank computerized health tracking systems in light of various factors.
- To extract and specify the relevant assessment factors for computer-based health monitoring systems. These factors might encompass significant parameters that affect the functionality and efficiency of such applications.
- To scrutinize the comparative significance of the selected evaluation criteria using the AHP. In order to achieve this, proficient-driven pairwise comparisons will be employed to ascertain the significance and weighting of each criterion. This approach will ensure that the appraisal procedure accurately reflects the preferences of all stakeholders.
- To prioritize computer-based health monitoring apps by using the ARAS approach while taking into account how well they perform in comparison to the weighted parameters. The use of ARAS was made it possible to evaluate applications thoroughly, which has eventually helped find the most suitable solutions.
- To validate and determine the hybrid MCDA model’s efficacy and integrity by contrasting its outcomes with those of other assessment techniques or expert opinions and to show further that using a hybrid method yields outcomes that are more accurate and reliable during the assessment of computerized health monitoring systems.
- To give healthcare experts the information they need in order to choose, deploy, and use technological health monitoring applications that will enhance patient care and healthcare performance.
- To advance the assessment processes of health technology by proposing a hybrid MCDA framework, thereby promoting more extensive and impartial assessment, which subsequently fosters modernization and excellence.

The research findings provide valuable insights that enable policymakers, technologists, and healthcare professionals to make well-informed decisions on the adoption and integration of computer-based health monitoring systems.

The sections followed in this research are as given in detail: The initial segment, referred to as the introduction, encompasses an overview of the intended study such as the contextual framework, the importance, and the aims of this research. The second section of this study provides a comprehensive review of the existing literature on health monitoring technology. The third segment delineates the research methodology utilized in the study and expounds upon the intricate steps entailed in the hybrid AHP and ARAS approach. In the fourth section, an analysis, interpretation, and discussion of the evaluation’s findings are undertaken. The fifth segment of the study provides a summary of the key discoveries, reiterates the research aims, and emphasizes the research’s contributions.

## 2 Literature review

The HAM10000 dataset, which consists of high-quality pictures of seven different types of skin disorders, was analyzed by Shahin et al. (6) using a combination of 16 different convolutional neural network simulations constructed using deep learning. Utilizing images of lesions as input, these algorithms were successful in correctly diagnosing and categorizing skin conditions. It takes time to determine skin problems in medical environments with inadequate assets. The tool for early skin-related illnesses identification is being developed to avoid unexpected health implications that might be expensive, take up valuable surgical time, and perhaps endanger patient health. The idea has changed throughout time, and contemporary investigation teams use it in a variety of covert situations. A statistically self-governing entity is referred to as an “agent” if it has the capacity to see, react to, and record actions taken by other agents and their surroundings. The analysis looks at the application and assessment of agent-based approaches in regard to a particularly significant problem. Infectious and non-transmissible illnesses, interpersonal and collaborative behavior, and social epidemiological studies are listed by Sulis et al. (7) as the main subjects of agent-based studies in the medical field. The study’s conclusion was reached after carefully reviewing significant prior research and performing an in-depth network assessment. The results are in line with an assessment of a number of prospective strategies for boosting the usage of agent-based methods in medicine.

The characteristics and intricacy of computer networks are always growing. Knowledge and data communication are crucial components of the healthcare system for the delivery of healthcare at all stages. Since accessibility and interaction are necessary for medical facilities to operate effectively, whether they are running independently or as a part of a network, the healthcare industry is critical to technological surveillance and crisis management. The proposed work (8) has three main goals: to define the qualities of an efficient monitoring approach; to evaluate the tracking options that exist in a multi-location medicine setting; and, lastly, to propose a thorough and widespread logical design for a surveillance system that can function in these circumstances. The proposed study looked at previous research on medical robots for old people using the paradigms of nursing, health, and human-computer interaction. Sophisticated robots employ mostly visual and auditory detectors and actuators that have restricted diagnostic capabilities, which makes them less beneficial than older generations. The findings highlight the necessity of a multidisciplinary research group that continuously works to improve the convenience and usefulness of automated devices for the care of old people (9).

The medical nursing subcategories have each been appropriately handled by the training sector using information approaches. The educational industry makes substantial use of network technology because of its usefulness, efficacy, and simplicity. The proposed research (10) initially provides a brief explanation of wireless technology, therapeutic surgery, and nursing principles before explaining how the three concepts may be merged. The specific study also examines how networking equipment is used in clinical learning for nurses. It first conducts research on and describes the qualities that need to be present throughout the phases of the computer and VR technology incorporation, such as composition qualities, knowledge traits, technological attributes, and practical abilities, before moving on to the features of Virtual Reality (VR) outcomes. The examination of stimulating VR input techniques and necessary VR equipment. Zhu et al. (11) provide information presentation tactics that make use of VR features after thoroughly defining the collaborative input aspects of computer and VR technologies. Then, utilizing tried-and-true graphic design methods, the variations and similarities between VR and traditional displays are contrasted. The findings of the study demonstrate that the experimental group outperformed the control group in terms of performance. VR technology in educational settings can thereby enhance student learning.

The most severe physical condition that can cause neurocognitive disability in children is cerebral palsy (CP), which causes mild to moderate dementia as well as persistent motor issues. Long-term disorder adversely impacts the level of life of CP children, their families, and ultimately society. A computerized language instruction program for children with CP is created using voice recognition. To improve CP rehab instruction, the motion-control feature of the bionic gripper may be integrated with HRI technology. The LabVIEW platform, which offers instantaneous fashion dynamic training and assessment of human and robotics motions and activities, was used to construct the human-machine communication elements. A person with CP could benefit from the training if they want to improve their reflexivity, verbal expression, or capability to discern movements from actions. Additionally, it offers crucial technologies for modifying the CP therapy course plan (12) and some other related studies (13–18, 37). Data management, storage, and collection methods have improved with the advancement of computer technology, and the area of data mining has expanded in response to the increase in demand. It is necessary to understand the theoretical underpinnings, the particular uses, and the environment in which these uses are now operating because technology usage in healthcare remains in its early stages. The primary areas of debate proposed by Zhi-Gen et al. (36) focus on the most recent advances in science as well as those that have recently occurred in the disciplines of DNA assessment, interpreting medical picture data, and the investigation of data gathered through the tracking of various physiological indicators.

One of the newest medical specialties is rehabilitation therapy. Among them, rehabilitative medical training is linked to both theory and real-world application and, by expertly melding the two, aids students in developing their practical skills. As a consequence of continuous shifts in the globe, computer multimedia infrastructure has developed and is now utilized in educational settings. Gao (22) has investigated conventional educational and instructional approaches in rehabilitation therapy in light of this. Next, it is discussed how computer multimedia techniques are used in healthcare instruction and education. Healthcare companies today employ centralized delivery mechanisms to offer superior treatment as a result of enhanced transparency. The proposed research (24) provides an overview of the current state of telemedicine. Telemedicine is one of the essential elements of computer-supported cooperative work (CSCW), a communications-based system. Computer-aided teleconferencing solutions are increasing the efficacy of remote medical care. To accomplish this, high-performance connectivity and fewer data transfers throughout the session could be employed. The design of a telemedicine system is illustrated.

The research thoroughly evaluates how computer technology is employed in society’s reaction to newly detected cases of crown influenza and recurring issues. It is dedicated to making the most of computer technology’s strengths and positive impacts, enhancing its capacity to assist in resolving social problems and utilizing technological devices to create a shared and cooperative global data network. It is both an inescapable direction in the big data era and a contemporary management tactic to use big data techniques and tools to control people (20). There have been instances involving both internal and external medical uses using computer vision technology. There is a huge possibility for meaningful patient advantage, particularly in the surgical setting, and there is a huge amount of data that may be used for statistical strategies. The goal of the study proposed by Kennedy-Metz et al. (27) is to provide a concise overview of the key computer vision concepts that are essential to the surgical field. The use of computer vision in operating rooms has many advantages, but efficiency and protection of patients are just two of them. Renowned specialists assert that there is relevant literature in the domains of surgical procedures, algorithmic evaluation and simulation in medicine, and visualization in medical care. Research findings encourage the use of computer vision technology during surgical procedures. The work suggests gathering and sharing well-annotated datasets to grow the subject.

An essential component of technology-based studies is the creation of an interactive smart blood transfusion set, that enables a computerized knowledge framework for a contemporary blood transfusion set solution and boosts the efficacy of smart blood transfusion tracking. The proposed research (25) indicated that the electrical supply wire was linked to the control interface, and a pharmaceutical delivery port was constructed on the side wall of the attaching head using computer modeling technology. Over the past 100 years, vocational nursing has grown in importance within the nursing profession. Every aspect that affects a worker’s health while they are at work is referred to as workplace wellness in its widest sense. Therapists and other medical personnel are working to build customer service programs at their places of employment that utilize advancing computer technologies. Integrated monitoring of illnesses in the context of workplace safety is one effective computer usage. McKenna (32) has examined the design, implementation, and assessment of an electronic health tracking system in a workplace wellness scenario.

The use of telephysiotherapy was asserted to raise the calibre of services provided by physiotherapists. Since the majority of visual criteria are used to assess the level of physiotherapy tasks, computer vision tools may be beneficial and valuable in system tracking. Kittipanya-ngam et al. (28) have addressed the possibility of tele-physiotherapy utilizing these advances and provide a case study of predicting falls through computer vision. The study’s findings suggest that further research is necessary before computer vision can be used to complete and improve telephysiotherapy technology. The potential uses of the novel interface layout for recorded virtual reality are the subject of numerous studies. Using this state-of-the-art semi-interactive technology, Lundstrom and Fernaeus (31) looked at two actual scenarios: elder care and professional psychological treatment for agoraphobia. The research groups approved the strategies since they worked effectively and were well-liked by both patients and professionals. These limited trials’ results stunned the study’s HCI specialists because they had such a tenuous connection to the language of VR as we understood it. The study examines the possible future contribution of the longstanding discipline of computational science to the subject of integrated virtual reality by considering the suggested structure procedure and observations from practical scenarios.

Computer technology may be used to alleviate some of the current challenges associated with rural medical care, including distance, a physician shortage, interaction, and knowledge cooperation. The investigation assesses recent system-design research and shares the system-design expertise in an effort to enhance patient consistency of care in remote regions. The recommended research has communicated with clinicians who practice in remote regions, visited the sites, and analyzed the present paper-based method in order to deeper comprehend the difficulties at hand and consequently offer a better plan to solve them (33). The daily burden for doctors and nursing personnel must be reduced in order to make optimal utilization of the resources available to them in healthcare facilities. This can only be done by increasing the efficacy of healthcare services and nursing tasks. Typically, clinical and nursing tasks are divided into five phases: surgical evaluation, clinical procedure, rehabilitation, and lifesaving care following rehabilitation. During these periods, training and learning are provided to healthcare and nursing specialists. Several robotic and computer-based technologies have been established throughout these phases to help with the tasks. For every phase, Gofuku (23) has offered a few implementation scenarios. The study then discussed two of their earlier pieces. One is a sophisticated virtual reality-based mirror visualization device for nearby pain syndrome. The next step involves performing a robotic Mendelsohn motion to preserve an older adult person’s usual ability for ingesting.

The statistical difficulties that arise in diagnostic procedures, treatment, and other therapeutic uses are thoroughly discussed. These encompass tracing immense, moving bacterial swarms, monitoring immune cell dispersal and recognition, and more. They also offer the most precise classification and assessment of 3D images of various medical items. The work illustrates how to apply novel approaches to see obstacles as mathematical issues. The proposed approaches are based on state-of-the-art architectural improvement, data mining, machine learning, and deep learning models and strategies. The investigation includes empirical results and suggestions that illustrate how these notions may be put to use in medical environments (35). Among the most important aspects of medicine is the identification of disorders such as liver fibrosis. The patient’s prior medical history is taken into account when determining if the following step is indeed required. Making the more appropriate decision at the right moment is never simple due to the condition’s fluctuating character and the concomitant haziness and ambiguity. Aswathy et al. (19) have addressed the issue of diagnosing liver sufferers which was previously raised. The suggested CNN framework is a 13 convolutional level, three completely linked level, SegNet-inspired VGG-16 encoder-decoder system. To investigate this, over 100 samples of liver imaging data were employed. The analysis also examines the suggested approach to a range of alternative classifier techniques. A precision of 98.3% was attained through training and assessment.

## 3 Methodology

Computer technology’s challenges and development remain constantly growing. The medical sector depends on data and knowledge exchange to provide services at all levels. The medical sector is particularly significant when considering computer systems monitoring and disaster prevention since connectivity and interaction are essential for the efficient functioning of medical units, whether they are working alone or as part of an interconnected system. The proposed article concentrates on applications of computer technology in healthcare systems. For this purpose, a feature-concentrated strategy for evaluation is designed to construct a reliable and accurate statistical process. The cornerstone of it is the characteristics outlined in the published works. A typical procedure for identifying and selecting an effective application is used in the assessment of computer-based medical surveillance apps. The second stage, which uses the AHP and ARAS methodologies, incorporates attributes relevant to computer-based health monitoring apps and makes choices after selecting the top-ranked application. This article is an in-depth review of the steps taken to provide the healthcare industry with the infrastructure needed for features-based screening for the efficient applications of computer technology in the healthcare sector. Finally, an accurate statistical analysis has been done and an effective application of computer technology in the healthcare sector has been determined in this article. Individuals are assisted in their efforts to handle challenges linked to energy by the use of MCDM approaches (26). An MCDM concern for the aviation industry is choosing the caterer. In order to select a caterer that will most effectively satisfy the demands of the airline, the screening procedure essentially comprises the examination of a variety of intricate parameters. There are multiple uses for the service vendor selection paradigm. The proposed study (21) approaches the challenge of selecting a catering vendor from the point of view of potential solutions. The AHP is paired with a specific outcome analysis in the proposed study, which comprises figuring out utility levels using ARAS and multi-selection target programming. A real-world scenario of picking a food vendor is also provided. The goals of Toygar et al. (34) were to investigate the problems brought on by a lack of storage space and to offer the best remedies. On the basis of data from the research, industrial papers, and specialist remarks, an MCDM framework that includes five parameters for identifying options and four primary factors, and 16 subgroups to characterize the challenges was presented. The graded ranking of priority for the concerns in the suggested framework was developed employing the SWARA (Step-wise Weight Assessment Ratio Analysis) methodology after the responses were assessed employing the ARAS technique. The study’s findings indicated that price rises, irregular supply chains, volume reduction, and a surge in blank sailing notices were the most pressing issues. Alternatives that were seen to be realistic included technological innovation, incentive calls, complete carrier services, and reservation guarantee uses.

### 3.1 Evaluation model architecture

**Figure 1:**
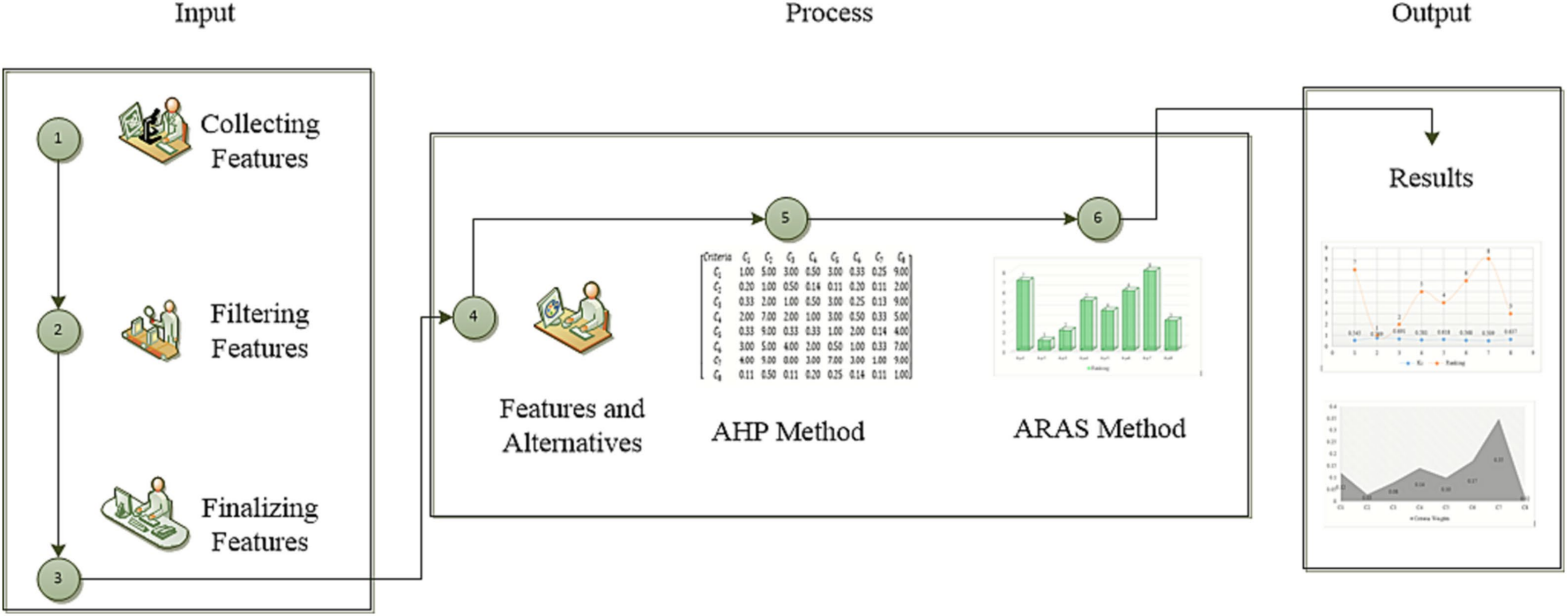
Evaluation framework.

The decision-making techniques are essential to evaluate the computer-based health monitoring applications and to select a suitable alternative among several options. To perform this task, hybrid MCDA-based AHP and ARAS techniques have been utilized as statistical procedure and selection techniques to identify an appropriate targeted alternative for healthcare sectors. Computer technology and health monitoring features have been categorized by the AHP prior to obtaining effective input from computer and healthcare scholars. The proposed assessment procedure has been categorized into three levels. The incorporation of the parameters into the screening process is the first level. The inherent characteristics of the options are ascertained at the second processing stage via AHP calculations. The sophisticated health monitoring application based on computer technology is selected utilizing the ARAS technique determined by the outcomes assigned for the benchmark after all available real-world data and expert views have been considered in the third level. The proposed assessment structure for the selection and statistical analysis of the computer-based health monitoring applications to digitalize and modernize the healthcare industry is as illustrated in Figure 1. It is founded on the use of the ARAS and AHP methodologies. The processes include in the proposed evaluation procedure are as discussed below in detail.

### 3.2 Features engineering

**Figure 2:**
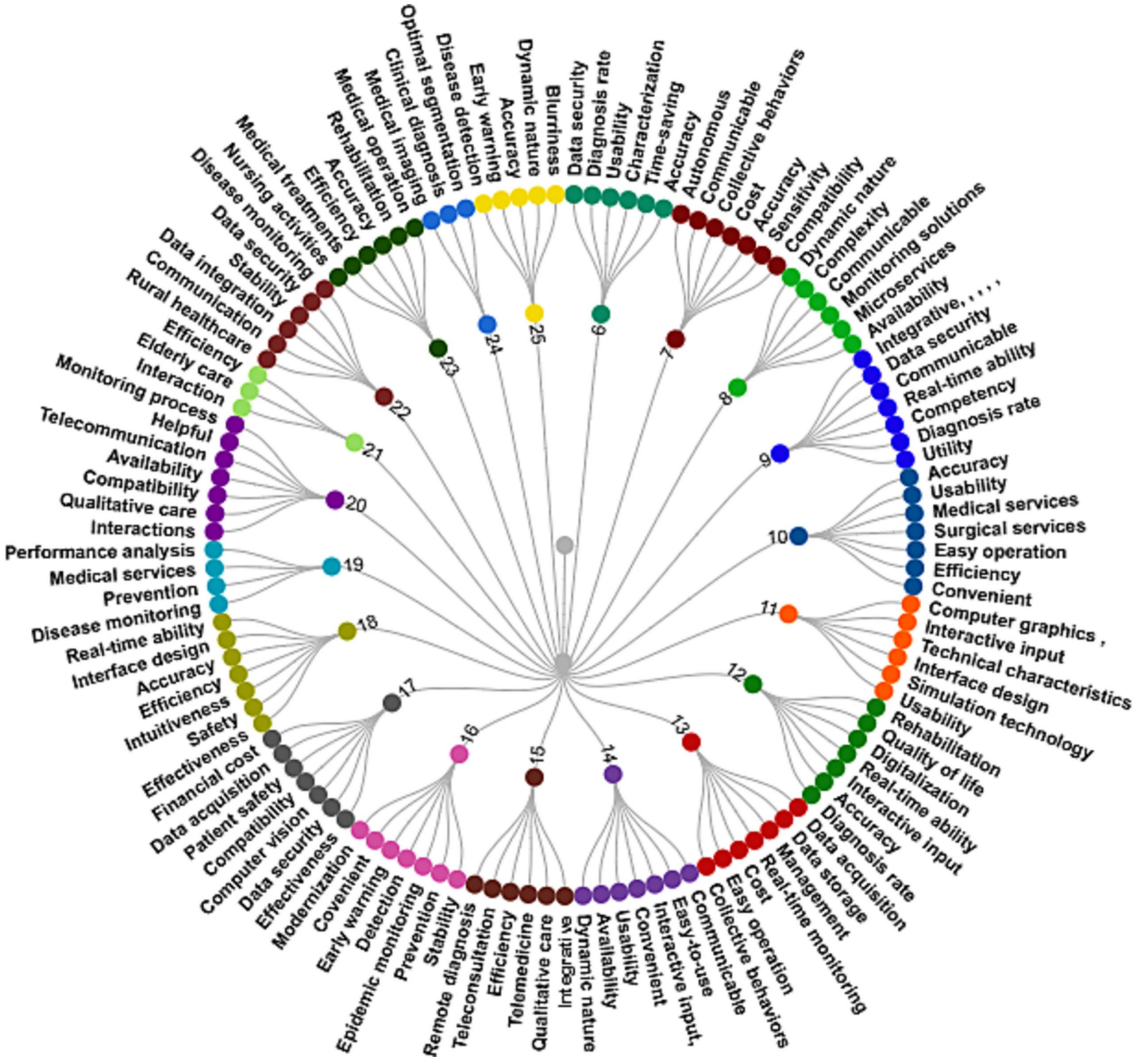
List of the extracted features.

The present statistical framework’s initial stage lays the greatest concentration on classifying parameters and options. The cornerstone of the suggested design is the development of comparing metrics for picking trustworthy computer technological applications to handle accuracy and performance issues in health monitoring settings. To ensure disease diagnosis accuracy and device performance, the attributes concentrating on detection and monitoring issues are extracted. The main argument in favor of using Safety precautions is that they cover every facet of computer technology. Computer technologies’ features are built from a multitude of libraries. To discover and collect the essential characteristics for contrasting computer applications incorporated into health monitoring systems, a thorough and detailed analysis of publications is carried out. These characteristics are relatively common since so many different detection and monitoring methods use them. There are now just eight (8) traits remaining beyond the initial comprehension of 121 features from various sources, enabling study and decision among many alternative applications. All of the characteristics from the academic research analysis are outlined in Figure 2.

The rationale behind employing the MCDA framework in the study stems from the requirement for an organized and methodical way to rank computer-based health monitoring apps. With the help of MCDA, decision-making in the healthcare industry may be handled with a strong approach that guarantees a careful and knowledgeable evaluation of the available options.

### 3.3 AHP technique

**Figure 3:**
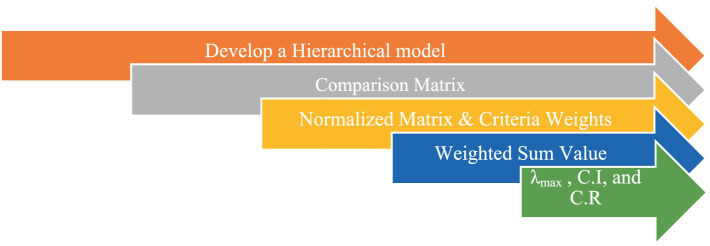
AHP steps.

This MCDA method is effective in structuring and investigating unpredictable choices. AHP provides a realistic approach for dealing with a number of challenges relating to numerous domains while making accurate and useful decisions. We may make informed decisions from the many alternatives that are available based on a variety of different aspects by using the Saaty scale that is offered. Saaty first proposed this technique in 1980 (29). To establish how much each characteristic is weighed, the present research uses the AHP technique for the selected parameters. To boost the efficiency of the healthcare industry, ARAS ranks a number of computer-based health monitoring applications using these weights. Every step of utilizing AHP to calculate the measures of the comparative parameters is as shown in Figure 3.

### 3.4 Step 1. Identifying features and alternatives

**Figure 4:**
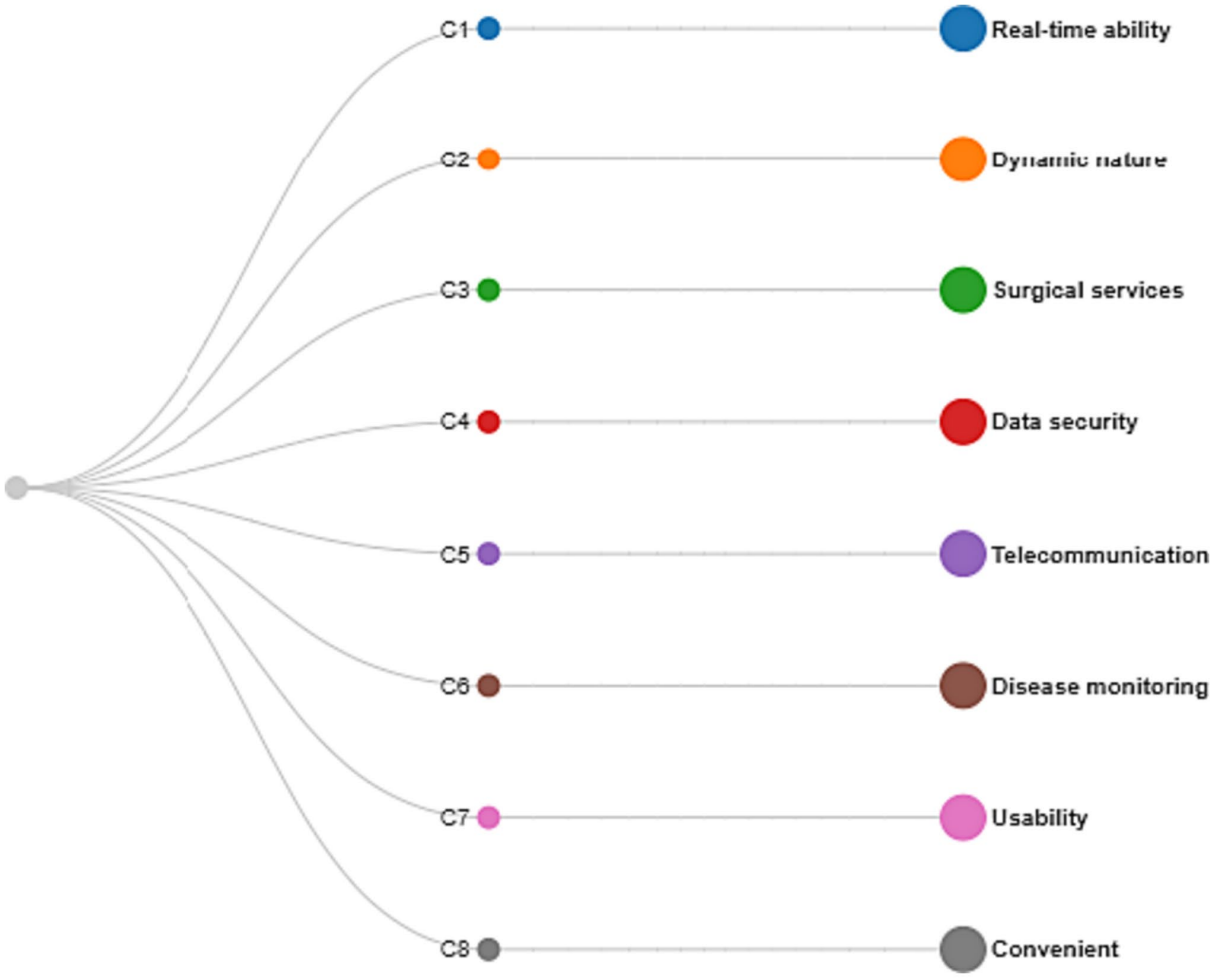
List of selected features.

The views and data from a computer scholars panel are structured and compared with eight adaptive applications such as Ap1…Ap8. The performance and preciseness of health monitoring technologies have been analyzed in a computer-based technological context. In terms of their stipulated benchmarks, only the alternative that received the strongest utility degree among all workable alternatives in light of the predefined features would be recognized as the ideal option. A set of eight (8) chosen features include real-time ability (C1), dynamic nature (C2), surgical services (C3), data security (C4), telecommunication (C5), disease monitoring (C6), usability (C7), and convenient (C8). It all relies on how they collaborate with each other to determine how efficacious the parameter is. A diagram that displays the criteria that were chosen was created by us. In Figure 4, the parameters can be seen as branches.

### 3.5 Step 2. AHP tree diagram

**Figure 5:**
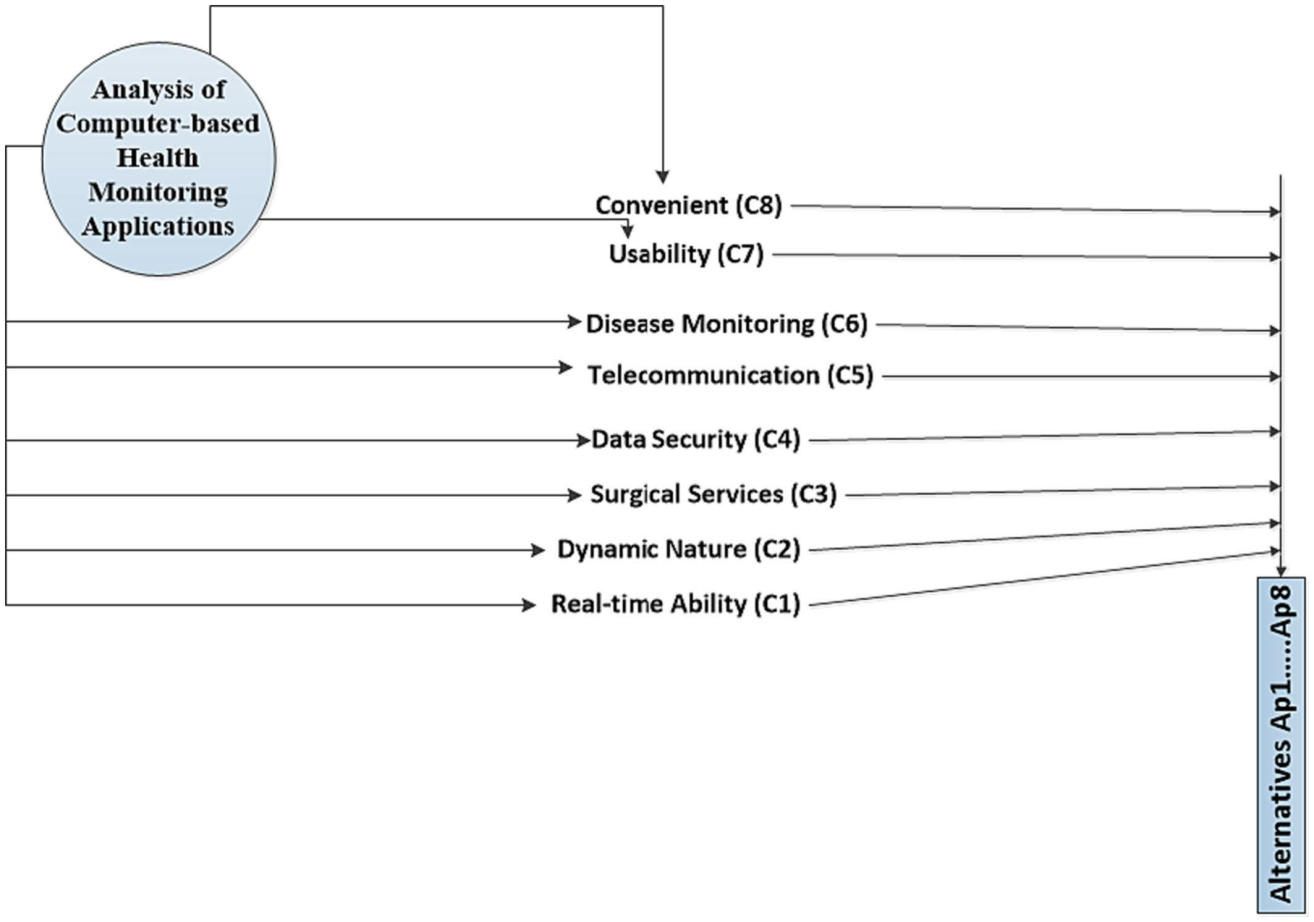
AHP hierarchical structure.

The decision-making dilemma is portrayed in three tire structural diagram that consists of a goal, criteria, and alternative in a tree shape architecture. Tire 1 illustrates the goal of the study. Tier 2 indicates a set of criteria chosen for analysis. While the last tier displays the alternatives that will be compared and assessed, as depicted in Figure 5.

### 3.6 Step 3. Construction of decision matrix

The diagrammatic designation is best for visual review even though it is not appropriate for computer modeling. Similarly to this, it becomes more complicated to grasp visually the attached image as the scheme becomes larger. It is crucial to offer an introduction that is simple to understand so that systems can keep, access, and assess data properly. In keeping with the Saaty measure, a matrix is produced by giving every attribute an assigned rating in line with the requirements of the specialist. In the end, an 8 8 pair-wise analysis matrix is generated and put together for the current circumstance. The matrix below shows the weight distribution of every parameter determined by the computer and medical professionals using a rating system.


$$\left[\begin{array}{ccccccccc} \mathrm{Criteria} & C_{1} & C_{2} & C_{3} & C_{4} & C_{5} & C_{6} & C_{7} & C_{8}\\ C_{1} & 1.00 & 5.00 & 3.00 & 0.50 & 3.00 & 0.33 & 0.25 & 9.00\\ C_{2} & 0.20 & 1.00 & 0.50 & 0.14 & 0.11 & 0.20 & 0.11 & 2.00\\ C_{3} & 0.33 & 2.00 & 1.00 & 0.50 & 3.00 & 0.25 & 0.13 & 9.00\\ C_{4} & 2.00 & 7.00 & 2.00 & 1.00 & 3.00 & 0.50 & 0.33 & 5.00\\ C_{5} & 0.33 & 9.00 & 0.33 & 0.33 & 1.00 & 2.00 & 0.14 & 4.00\\ C_{6} & 3.00 & 5.00 & 4.00 & 2.00 & 0.50 & 1.00 & 0.33 & 7.00\\ C_{7} & 4.00 & 9.00 & 8.00 & 3.00 & 7.00 & 3.00 & 1.00 & 9.00\\ C_{8} & 0.11 & 0.50 & 0.11 & 0.20 & 0.25 & 0.14 & 0.11 & 1.00 \end{array}\right]$$

### 3.7 Step 4. Normalizing a decision matrix and criteria weights (CW)

The constructed pairwise comparison matrix has been normalized using Eq. (1) to remove subjectivity from them. The process indicates that every individual criterion value was divided by the total of every column for obtaining the normalized scores. The identified normalized matrix is as shown below;


(1) $${\bar{X}}_{ij}=\frac{X_{ij}}{\mathrm{Sum}\;\mathrm{of}\;\mathrm{every}\;\mathrm{column}}$$

After determining the normalized matrix, we then apply Eq. (2) on it to calculate the relative importance of every criterion. The process indicates that the sum of every row was divided by the total number of criteria and calculated the relative weights of criteria, as shown below;


(2) $$\mathrm{Crieria}\;\mathrm{Weights}=\frac{\sum {\bar{\bar{X}}}_{ij}}{\mathrm{Total}\;\mathrm{number}\;\mathrm{of}\;\mathrm{criteria}}$$

$$\left[\begin{array}{cccccccccc} \mathrm{Criteria} & C_{1} & C_{2} & C_{3} & C_{4} & C_{5} & C_{6} & C_{7} & C_{8} & CW\\ C_{1} & 0.09 & 0.13 & 0.16 & 0.07 & 0.17 & 0.04 & 0.10 & 0.20 & 0.12\\ C_{2} & 0.02 & 0.03 & 0.03 & 0.02 & 0.01 & 0.03 & 0.05 & 0.04 & 0.03\\ C_{3} & 0.03 & 0.05 & 0.05 & 0.07 & 0.17 & 0.03 & 0.05 & 0.20 & 0.08\\ C_{4} & 0.18 & 0.18 & 0.11 & 0.13 & 0.17 & 0.07 & 0.14 & 0.11 & 0.14\\ C_{5} & 0.03 & 0.23 & 0.02 & 0.04 & 0.06 & 0.27 & 0.06 & 0.09 & 0.10\\ C_{6} & 0.27 & 0.13 & 0.21 & 0.26 & 0.03 & 0.13 & 0.14 & 0.15 & 0.17\\ C_{7} & 0.36 & 0.23 & 0.42 & 0.39 & 0.39 & 0.40 & 0.42 & 0.20 & 0.35\\ C_{8} & 0.01 & 0.01 & 0.01 & 0.03 & 0.01 & 0.02 & 0.05 & 0.20 & 0.02\\ \mathrm{Sum} & 1 & 1 & 1 & 1 & 1 & 1 & 1 & 1 & 1 \end{array}\right]$$

To determine the weighted normalized matrix, we then multiply the calculated criterion weights with the normalized matrix, as shown below;


$$\left[\begin{array}{cccccccccc} \mathrm{Criteria} & C_{1} & C_{2} & C_{3} & C_{4} & C_{5} & C_{6} & C_{7} & C_{8} & CW\\ C_{1} & 0.09 & 0.13 & 0.16 & 0.07 & 0.17 & 0.04 & 0.10 & 0.20 & 0.12\\ C_{2} & 0.02 & 0.03 & 0.03 & 0.02 & 0.01 & 0.03 & 0.05 & 0.04 & 0.03\\ C_{3} & 0.03 & 0.05 & 0.05 & 0.07 & 0.17 & 0.03 & 0.05 & 0.20 & 0.08\\ C_{4} & 0.18 & 0.18 & 0.11 & 0.13 & 0.17 & 0.07 & 0.14 & 0.11 & \times \;0.14\\ C_{5} & 0.03 & 0.23 & 0.02 & 0.04 & 0.06 & 0.27 & 0.06 & 0.09 & 0.10\\ C_{6} & 0.27 & 0.13 & 0.21 & 0.26 & 0.03 & 0.13 & 0.14 & 0.15 & 0.17\\ C_{7} & 0.36 & 0.23 & 0.42 & 0.39 & 0.39 & 0.40 & 0.42 & 0.20 & 0.35\\ C_{8} & 0.01 & 0.01 & 0.01 & 0.03 & 0.01 & 0.02 & 0.05 & 0.20 & 0.02 \end{array}\right]$$

### 3.8 Step 5. Weighted sum value, and ratio of weighted sum value and criteria weights

The outputs obtained from the above multiplication process were obtained and then, the total of every row was then calculated, which is known as the weighted sum value of each criterion. The ratio between criterion weights and sum value has been calculated using a process in which the weighted sum value was divided by the criterion weights. The entire process of this step is as shown in Table 1.

**Table 1:** Weighted sum value and ratio between weighted sum value and CW.

Criteria	Weighted sum value	CW	Weighted sum value / CW
C1	1.18	0.12	9.88
C2	0.23	0.03	8.78
C3	0.80	0.08	9.88
C4	1.32	0.14	9.75
C5	0.91	0.10	9.14
C6	1.56	0.17	9.38
C7	3.50	0.35	9.92
C8	0.17	0.02	8.70

### 3.9 Step 6. Computation process of $\lambda_{\text{max}}$ , CI, and CR

As we know that Eq. (3);


(3) $$\lambda_{\text{max}}=\frac{9.88+8.78+9.88+9.75+9.14+9.38+9.92+8.70}{8}$$

The output of CI (Consistency Index) was computed using Eq. (4);


(4) $$CI=\frac{\lambda_{\text{max}}-n}{n-1}$$

while n represents the number of attributes/criteria.

By putting the values, the equation becomes;


$$CI=\frac{9.43-8}{8-1}$$

CI=0.20

The provided formula has been implemented to identify the Consistency Ratio (CR) as follows Eq. (5);


(5) $$CR=\frac{CI}{RI}$$

Whereas the score of RI (Random Index) at the 8th position is 1.41, the equation becomes;


$$CR=\frac{0.20}{1.41}$$

CR=0.1

**Figure 6:**
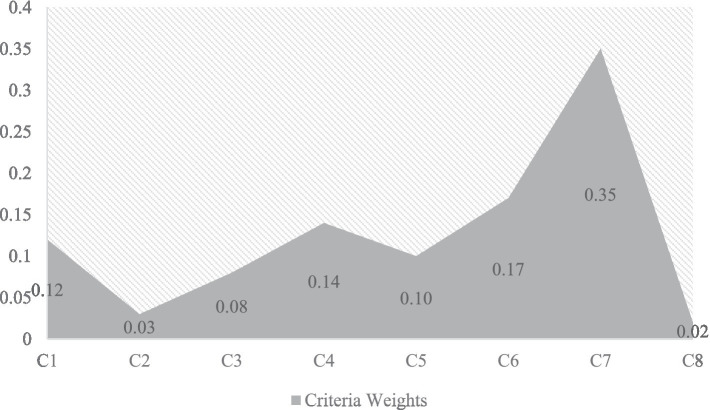
Criteria weights.

Graphical representation is an appropriate and well-matched representation way for visual analysis. All the criterion weights calculated using the AHP approach are presented in graphical form to easily comprehend by the readers, as shown in Figure 6.

### 3.10 ARAS technique

**Figure 7:**
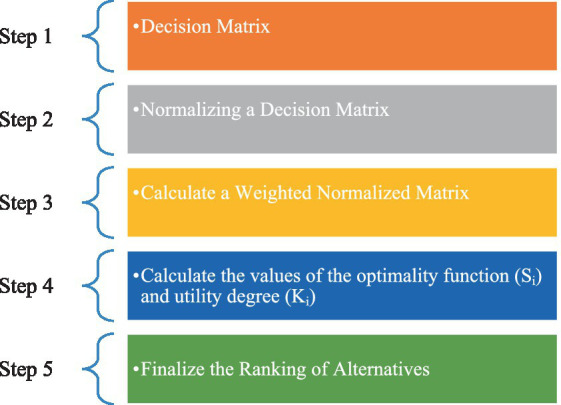
ARAS steps.

Numerous industries rely on the ARAS methodology to aid in their decision-making processes. By simplifying intricate decision-making scenarios, the ARAS technique picks the precise solution. The comparative indicator is capable of highlighting disparities between the suggested course of action and the options at hand. This is attained without the effects of diverse measuring units from the equation (30). For computer and healthcare specialists to make the best decisions, the ARAS statistical analysis approach combines the usage of weights and parameter additions throughout the data processing stage. This method acts as an assessment and statistical equipment to decide on the best-designed choice for a reliable and efficient computer-based health monitoring mechanism. Computer specialists’ hiring and the use of AHP for classifying performance- and monitoring-related factors have both already been discussed. The chosen attributes of the top-ranking competitor are then used to choose which computer application to use as the one to be targeted. An exemplification of the procedural stages entailed in the approach employed to scrutinize and prioritize the proposed blueprint for health monitoring applications that operate on computer technology is portrayed in Figure 7. This section furnishes data pertaining to the stages and specifics of the ARAS with respect to alternatives.

### 3.11 Step 1. Decision matrix representing alternatives and features

The usage of visual representations is most suitable for the purpose of visual selection, although it may not be conducive to computer processing. Moreover, the complexity of the connected graph increases pointedly for larger systems, thereby affecting its pictorial clarity. Thus, it is imperative to develop a straightforward paradigm that assists proficient data gathering, availability, and analysis by machines. The identified feature exhibits advantageous features in this context. An 8 × 8 decision matrix has been created to present the performance related attributes and data collected from specialists in both computer and healthcare environments. The decision matrix has been designed to include eight options for selection, based on the defined attributes, featuring eight factors arranged in columns. The identification of the optimal value (OV) in each column has been established. The perspectives gathered from computer experts have been categorized into eight distinct options based on the weights assigned to each attribute, as illustrated in the matrix below.


$$\left[\begin{array}{ccccccccc} \frac{\mathrm{Criteria}}{\mathrm{Alternatives}} & C_{1} & C_{2} & C_{3} & C_{4} & C_{5} & C_{6} & C_{7} & C_{8}\\ Ap_{1} & 4 & 6 & 9 & 3 & 5 & 8 & 2 & 7\\ Ap_{2} & 7 & 3 & 2 & 4 & 6 & 5 & 9 & 8\\ Ap_{3} & 2 & 4 & 6 & 7 & 5 & 3 & 8 & 4\\ Ap_{4} & 5 & 7 & 3 & 2 & 4 & 8 & 5 & 2\\ Ap_{5} & 3 & 2 & 5 & 6 & 2 & 4 & 7 & 9\\ Ap_{6} & 9 & 8 & 4 & 3 & 6 & 7 & 2 & 5\\ Ap_{7} & 2 & 6 & 7 & 5 & 4 & 6 & 3 & 2\\ Ap_{8} & 6 & 5 & 4 & 6 & 3 & 2 & 7 & 8\\ OV & 9 & 8 & 9 & 7 & 6 & 8 & 9 & 9 \end{array}\right]$$

### 3.12 Step 2. Normalized decision matrix

The decision matrix has undergone normalization with regard to both advantageous and disadvantageous parameters. The decision matrix elicits a desire for higher scores because of its entirely advantageous characteristics. The formulae shown below are used to normalize the parameters that are advantageous or disadvantageous.

For advantageous criteria as follows Eq. (6);


(6) $${\bar{X}}_{ij}=\frac{X_{ij}}{\sum_{i=0}^{m}X_{ij}}$$

For non-advantageous criteria as follows Eq. (7);


(7) $$X_{ij}=\frac{1}{X_{ij}};\;{\bar{X}}_{ij}=\frac{X_{ij}}{\sum_{i=0}^{m}X_{ij}}$$

The application of Eq. (6) facilitated the normalization process of the decision matrix, leading to the elimination of any subjectivity present, thanks to the advantageous nature of the parameters. The next step will be to multiply criterion weights derived from AHP by the resultant normalized matrix to obtain a weighted matrix. The calculated normalized values are as delineated in Table 2.

**Table 2:** Normalized decision matrix.

Criteria alternatives	C1	C2	C3	C4	C5	C6	C7	C8
Ap1	0.09	0.12	0.18	0.07	0.12	0.16	0.04	0.13
Ap2	0.15	0.06	0.04	0.09	0.15	0.10	0.17	0.15
Ap3	0.04	0.08	0.12	0.16	0.12	0.06	0.15	0.07
Ap4	0.11	0.14	0.06	0.05	0.10	0.16	0.10	0.04
Ap5	0.06	0.04	0.10	0.14	0.05	0.08	0.13	0.17
Ap6	0.19	0.16	0.08	0.07	0.15	0.14	0.04	0.09
Ap7	0.04	0.12	0.14	0.12	0.10	0.12	0.06	0.04
Ap8	0.13	0.10	0.08	0.14	0.07	0.04	0.13	0.15
OV	0.19	0.16	0.18	0.16	0.15	0.16	0.17	0.17
				×				
Criteria weights	0.12	0.03	0.08	0.14	0.10	0.17	0.35	0.02

### 3.13 Step 3. Weighted normalized matrix

The weighted matrix is obtained by multiplying the values of the parameters acquired through AHP with every parameter of the normalized matrix by applying the Eq. (8). The findings of this computation are showcased in a table presentation, as exemplified in Table 3.


(8) $${\hat{X}}_{ij}={\bar{X}}_{ij}*W_{j}$$

**Table 3:** Weighted normalized matrix.

Criteria alternatives	C1	C2	C3	C4	C5	C6	C7	C8
Ap1	0.010	0.003	0.015	0.009	0.012	0.026	0.014	0.003
Ap2	0.018	0.002	0.003	0.013	0.015	0.016	0.061	0.003
Ap3	0.005	0.002	0.010	0.022	0.012	0.010	0.054	0.001
Ap4	0.013	0.004	0.005	0.006	0.010	0.026	0.034	0.001
Ap5	0.008	0.001	0.008	0.019	0.005	0.013	0.047	0.003
Ap6	0.023	0.004	0.007	0.009	0.015	0.023	0.014	0.002
Ap7	0.005	0.003	0.012	0.016	0.010	0.020	0.020	0.001
Ap8	0.015	0.003	0.007	0.019	0.007	0.007	0.047	0.003
OV	0.023	0.004	0.015	0.022	0.015	0.026	0.061	0.003

### 3.14 Step 4. Determining optimality function (S_i_) and utility degree (K_i_)

The outputs of the optimality function, designated as P_i_, for every alternative, were derived by the utilization of the Eq. (9). This process was done by calculating the sum of every row of the weighted matrix.


(9) $$S_{i}=\overset{n}{\underset{J=1}{\sum }}{\hat{X}}_{ij};i=\bar{0,m}.$$

### 3.15 For the determination of the utility degree (K_i_)

The assessment of alternative solutions in relation to the Eq. (10) results in the determination of K_i_ for varying options.


(10) $$K_{i}=\frac{S_{i}}{S_{0}}$$

Whereas the output of P_o_ is 0.169 as well.

The outcomes derived from these equations have been delineated in Table 4.

**Table 4:** Calculation of S_i_ and K_i_.

Alternatives	S_i_	K_i_	Ranking
	0.169	1	
Ap1	0.092	0.545	7
Ap2	0.130	0.769	1
Ap3	0.117	0.691	2
Ap4	0.098	0.581	5
Ap5	0.104	0.618	4
Ap6	0.096	0.568	6
Ap7	0.086	0.509	8
Ap8	0.108	0.637	3

### 3.16 Step 5. Ranking of alternatives

**Figure 8:**
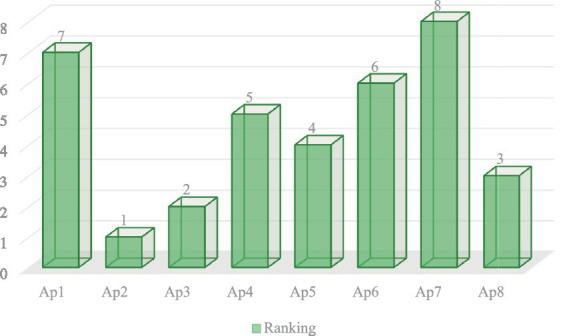
Alternatives ranking based on ARAS.

Based on the K_i_ output, the available alternatives have been painstakingly arranged in ascending order, with the most favorable being the one with the highest output and the least appropriate being the one with the lowest output. Figure 8 provides an illustration of the grading of alternatives that were identified by the utilization of the ARAS method.

## 4 Results and discussion

Numerous sections of the medical and healthcare industries have adopted the use of advanced technologies, particularly computer technology, and robotic equipment. Numerous opinions exist on the improvement of medical procedures and nursing procedures through the adoption of cutting-edge technologies. One of these opinions focuses on how medical systems based on state-of-the-art technology have developed to allow for the development of medical treatment alternatives and monitoring of the patient’s health on a real-time basis. Additionally, improving the effectiveness of medical procedures and nursing practices is essential for minimizing the regular chores performed by nurses and physicians and maximizing the use of healthcare and nursing home personnel. It is undeniable that computer applications will play a crucial role in today’s medical fields as long as computer technology improves. To achieve the essential outcomes, we develop a hybrid MCDA framework that precisely assesses computerized health monitoring applications and determines an appropriate application for the enhancement and preciseness of healthcare outcomes and patient care. The key objective of our research is to properly analyze and prioritize the computerized health monitoring applications against the numerous essential weighted factors.

**Figure 9:**
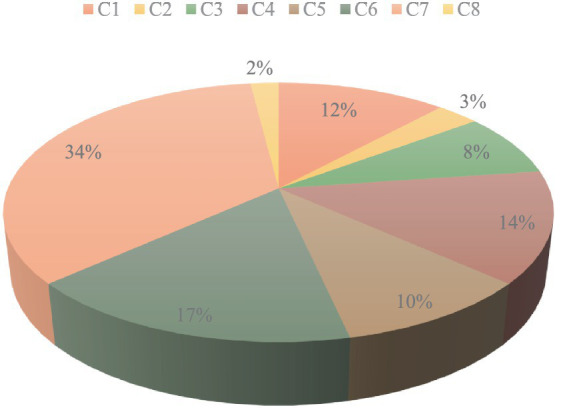
Criterion weights identified by AHP.

In this study, relevant assessment criteria for computer-based health monitoring apps were found and defined. A thorough set of comparative features was constructed by a thorough examination of the available literature and discussions with specialists in the subject. These features were used as a base for the subsequent assessment procedures and were thought to be essential for assessing the usability and efficacy of the applications. The AHP was then used to determine the respective weights of the determined assessment criteria. Pairwise comparisons were conducted with a group of physicians and technology specialists to ascertain the significant nature of every factor. The AHP analysis generated a collection of weights that were prioritized and expressed the values and intentions of the decision-making individuals. The use of criterion weights enabled a straightforward and data-driven way of analyzing the applications, which improved the assessment framework’s validity and objectivity. The outcomes determined by the AHP indicate that usability (C7) has the most higher weighted criteria with a score of 0.35, followed by disease monitoring (C6) with a weighted value of 0.17, data security (C4) with a weighted output of 0.14, real-time ability (C1) with a value of 0.12, telecommunication (C5) with a weight of 0.10, surgical services (C3) with a weighted score of 0.08, dynamic nature (C2) with a weight of 0.03, and convenient (C8) has the least weighted criteria with the lowest output of 0.02, as shown in Figure 9. These results demonstrate that the usability feature has the best-performing feature among the entire set of chosen features.

**Figure 10:**
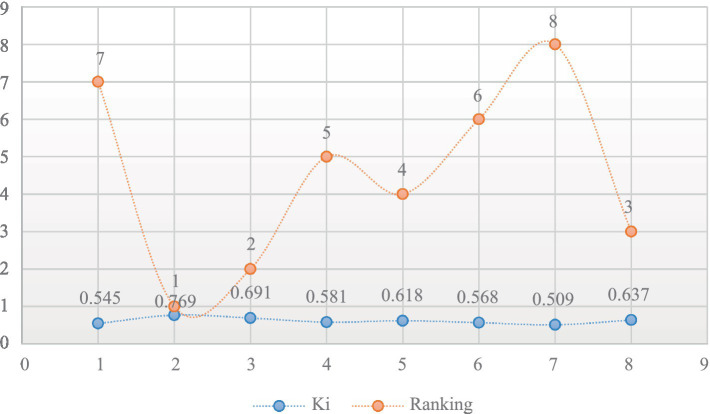
Ranking of alternatives determined by ARAS.

After weighing the chosen factors based on the AHP, the computerized health monitoring apps were then ranked on the basis of the efficiency with which they performed in comparison to weighed factors using the ARAS approach. This method made it easier to analyze every application thoroughly, which made it possible to pinpoint the best and most suitable solution. The utility degree values and ranking of each alternative determined by the ARAS indicate that the Ap2 has performed well against the weighted factors with an outcome of 0.769 and known as the best application, followed by the Ap3 with an output of 0.691 and secured 2nd place, Ap8 positioned at 3rd with a score of 0.637, Ap5 positioned at 4th with a value of 0.618, Ap4 positioned at 5th with a result of 0.581, Ap positioned at 6th with an output of 0.568, Ap1 placed at 7th with a degree of 0.545, and the last one Ap7 has performed negatively and worst against the weighted factors with the lowest value of 0.509 and known as the worst application in the entire set of alternatives, as shown in Figure 10. The entire set of assessed applications has been arranged in sequential order such as the alternative with the highest value placed in first place followed by the other alternatives in ascending order. The examination and ranking of the applications offered insightful views into their relative efficacy in accomplishing the chosen factors. We utilized the ARAS rankings as a unified yardstick for determining the apps’ general efficacy and performance.

The proposed research further contrasted the hybrid MCDA model’s findings with those acquired from other well-known ways of assessing or expert analyses in order to verify the methodology’s performance and accuracy. The comparison’s outcomes showed that the hybrid technique regularly generated precise and relevant outcomes, demonstrating its appropriateness for assessing computerized health monitoring systems. As a result of the verification procedure, the integrated MCDA model’s ability to provide a rigid and unbiased assessment mechanism has been shown effective, supporting its reputation as a decisive tool. These results have provided users, administrators, and physicians to participate in the integration and utilization of computerized health monitoring applications with rational decision-making assistance. For analyzing health monitoring apps, the integrated MCDA paradigm has provided a rigorous, data-focused approach. Making enlightened judgments has been made possible because of this. The scheme has also taken into account different criteria and stakeholder preferences, which makes it easier to choose apps that are most suitable for achieving certain healthcare targets and demands.

In addition, the findings of the research revealed the relevance of using technology-centric techniques for reviewing healthcare. The integrated MCDA paradigm has shown its ability for supporting inventiveness, enhancing the appraisal of health technology, and facilitating the incorporation of cutting-edge applications into the healthcare environments. It is simple to streamline and enhance computerized health monitoring applications thanks to the precise and targeted assessment procedure, which results in improved patient care and healthcare performance. The proposed study not only suggested multiple directions for additional studies but also demonstrated the effectiveness of the blended MCDA paradigm in the assessment of computer-based health monitoring applications. Additional research is necessary to determine whether incorporating cutting-edge technologies like AI and machine learning can improve the framework’s ability for customized and prescriptive health monitoring. The enduring effects of such applications on patient well-being and medical delivery may also be discovered through prospective research and real-world configurations, which may offer crucial novel knowledge.

All of the weights of the factors that were determined utilizing the AHP are exhibited in a visual format, depicted in Figure 9, to facilitate comprehension for the readers.

Figure 10 presents an elucidation of the classification of available options that were ascertained through the implementation of the ARAS methodology.

## 5 Conclusion

The utilization of computers is crucial to contemporary healthcare, life sciences, medical imaging, clinical diagnostics, human genome studies, and health information administration, among other areas. The importance of computer technology applications in today’s medical and healthcare is evident given the rapid growth of the use of computers. The increased usage and complex nature of the recently launched computer apps in the health sector have created hurdles for physicians and decision-makers to choose the most beneficial and acceptable solutions, customized to their particular requirements. The major purpose of our research was to create a thorough and impartial appraisal framework that used the statistical concept of the AHP and ARAS approaches to solve the evaluation and decision-making dilemma in a health monitoring environment. The investigation established a thorough analysis of the applications’ efficacy and utility by extracting and defining pertinent assessment factors such as real-time ability, surgical services, dynamic nature, data security, telecommunication, disease monitoring, usability, and convenience. Stakeholder-driven decisions were enabled through the use of AHP for evaluating the proportional importance of the chosen assessment standards while making sure that the screening process demonstrated the demands and targets of physicians and decision-makers. The ARAS technique is being used to rank applications based on how well they performed in relation to the assessed criteria, underscoring the design’s potential to deliver a thorough and comparable review. This ranking system makes it easier to identify the most useful and relevant apps, assisting medical experts in making the best selections for embedding health monitoring technology into their daily work. The findings determined by the developed MCDA framework show that the usability feature has the highest weight value of 0.35 among any other features. On the other side, the alternative Ap2 has the largest utility degree score of 0.769, which shows that this is an ideal and most favorable application by physicians and experts, while the alternative Ap7 has the minimum utility degree value, which demonstrates that this is least preferable by the medical experts and decision-makers. The hybrid MCDA framework’s performance and reliability have been confirmed by comparisons to other available evaluation methods and expert reviews. The outcomes illustrate that the system consistently produces precise and valuable outcomes, highlighting its appropriateness for the evaluation of computerized health monitoring systems. By utilizing research-based assistance for decision-making in this study, doctors, nurses, administrators, and stakeholders may be better equipped to make knowledgeable decisions, enhancing patient care and healthcare results. Doctors and nurses may more efficiently maximize incorporating and embracing innovative technologies via the use of an integrated MCDA system, improving the general caliber of healthcare operations. The hybrid MCDA paradigm is a useful tool for stimulating creativity, promoting quality in the evaluation of health technology, and making it simpler to integrate cutting-edge technologies into clinical practices. In conclusion, this study illustrates the essential significance of using MCDA-based AHP and ARAS procedures to ensure an in-depth and data-oriented analysis of computer-based health monitoring uses. It also points out the essential function of rational decisions in the domain of healthcare. The outcomes of the research will have a significant impact on decision-making and the potential use of technology-enabled medical services as the healthcare industry progresses. The cooperative efforts of Medical workers, scientists, and those in power will continue to determine healthcare trends, utilizing technology’s ability to enhance patient well-being and change the healthcare landscape for future generations.

## Data availability statement

The raw data supporting the conclusions of this article will be made available by the authors, without undue reservation.

## Author contributions

WHo: Conceptualization, Formal analysis, Methodology, Software, Writing – original draft, Writing – review & editing. GJ: Conceptualization, Data curation, Formal analysis, Investigation, Resources, Validation, Visualization, Writing – original draft. WHa: Conceptualization, Data curation, Formal analysis, Resources, Software, Writing – original draft. LW: Conceptualization, Data curation, Formal analysis, Funding acquisition, Project administration, Resources, Validation, Visualization, Writing – original draft, Writing – review & editing. MY: Conceptualization, Data curation, Formal analysis, Investigation, Methodology, Resources, Software, Supervision, Validation, Visualization, Writing – original draft, Writing – review & editing. LX: Funding acquisition, Project administration, Supervision, Writing – review & editing.

## References

[1] Sundström C Blankers M Khadjesari Z. Computer-based interventions for problematic alcohol use: a review of systematic reviews. Int J Behav Med. (2017) 24:646–58. doi: 10.1007/s12529-016-9601-8, PMID: 27757844 PMC5608865

[2] Menychtas A Doukas C Tsanakas P Maglogiannis I. (2017). A versatile architecture for building IoT quantified-self applications. IEEE 30th International Symposium on Computer-Based Medical Systems (CBMS). 500–505.

[3] Nguyen HH Mirza F Naeem MA Nguyen M. (2017). A review on IoT healthcare monitoring applications and a vision for transforming sensor data into real-time clinical feedback. IEEE 21st International Conference on Computer Supported Cooperative Work in Design (CSCWD). 257–262.

[4] Kemis H Bruce N Wang P Antonio T Lee Byung G Hoon Jae L. (2012). Healthcare monitoring application in ubiquitous sensor network: design and implementation based on pulse sensor with arduino. 2012 6th International Conference on New Trends in Information Science, Service Science and Data Mining (ISSDM2012). 34–38.

[5] Sharma A Prasad KDV Chakrasali SV Gowda VD Kumar C Chaturvedi A. Computer vision based healthcare system for identification of diabetes & its types using AI. Measurement. (2023) 27:100751. doi: 10.1016/j.measen.2023.100751

[6] Shahin M Chen FF Hosseinzadeh A Khodadadi Koodiani H Shahin A Ali NO. A smartphone-based application for an early skin disease prognosis: towards a lean healthcare system via computer-based vision. Adv Eng Inform. (2023) 57:102036. doi: 10.1016/j.aei.2023.102036

[7] Sulis E Mariani S Montagna S. A survey on agents applications in healthcare: opportunities, challenges and trends. Comput Methods Prog Biomed. (2023) 236:107525. doi: 10.1016/j.cmpb.2023.107525, PMID: 37084529

[8] Marques C Ramos V Peixoto H Machado J. Pervasive monitoring system for services and servers in healthcare environment. Procedia Comp Sci. (2022) 201:720–5. doi: 10.1016/j.procs.2022.03.097

[9] Dino MJS Davidson PM Dion KW Szanton SL Ong IL. Nursing and human-computer interaction in healthcare robots for older people: an integrative review. Int J Nurs Stud Adv. (2022) 4:100072. doi: 10.1016/j.ijnsa.2022.100072 PMC11080351 38745638

[10] Li X Zhao L. (2021). The application of computer network Technology in Surgical Nursing Teaching. 2021 International Conference on Education, Information Management and Service Science (EIMSS). 434–437.

[11] Zhu L Li Y Bai L Yu Q Li M. (2022). The development of VR technology and computer technology integration application. 2022 International Symposium on Advances in Informatics, Electronics and Education (ISAIEE). 476–481.

[12] Yu J Wang Y. (2022). Research on the application of computer virtual reality Technology in the Rehabilitation of special children. 2022 IEEE International Conference on Advances in Electrical Engineering and Computer Applications (AEECA). 1298–1302.

[13] He B Dai C Lang J Bing P Tian G Wang B. A machine learning framework to trace tumor tissue-of-origin of 13 types of cancer based on DNA somatic mutation. Biochimica et Biophysica Acta. (2020) 1866:165916 32771416 10.1016/j.bbadis.2020.165916

[14] He B Lang J Wang B Liu X Lu Q He J. TOOme: a novel computational framework to infer cancer tissue-of-origin by integrating both gene mutation and expression. Front Bioeng Biotechnol. (2020) 8:394. doi: 10.3389/fbioe.2020.00394, PMID: 32509741 PMC7248358

[15] He B Lu Q Lang J Yu H Peng C Bing P. A new method for CTC images recognition based on machine learning. Front Bioeng Biotechnol. (2020) 8:897. doi: 10.3389/fbioe.2020.00897 32850745 PMC7423836

[16] Lu S Yang J Yang B Yin Z Liu M Yin L. Analysis and Design of Surgical Instrument Localization Algorithm. CMES-Comp Mod Eng Sci. (2023) 137

[17] Tian F Pan J. Hospital bed supply and inequality as determinants of maternal mortality in China between 2004 and 2016. Int J Equity Health. (2021) 20:1–15. doi: 10.1186/s12939-021-01391-9 33516208 PMC7846917

[18] Wang W Qi F Wipf D Cai C Yu T Li Y. (2023). Sparse Bayesian learning for end-to-end EEG decoding. *IEEE Transactions on Pattern Analysis and Machine Intelligence*. 10.1109/TPAMI.2023.3299568 37506000

[19] Aswathy SU Ajesh F Shamsudheen S Jarin T. Computer-aided diagnosis of liver fibrosis in hepatitis patients using convolutional neural network. Comp Anal Deep Learn Med Care. (2021):217–36. doi: 10.1002/9781119785750.ch9

[20] Ding Y Jiang Y. (2021). Analysis on the application of computer information Technology in the Community's response to the COVID-19-- take big data as an example. 2021 International Conference on Computers, Information Processing and Advanced Education (CIPAE). 201–204.

[21] Fu Y-K. An integrated approach to catering supplier selection using AHP-ARAS-MCGP methodology. J Air Transp Manag. (2019) 75:164–9. doi: 10.1016/j.jairtraman.2019.01.011

[22] Gao P. (2021). Application of computer multimedia Technology in the Field of rehabilitation medicine training. 2021 2nd International Conference on Smart Electronics and Communication (ICOSEC). 1001–1004.

[23] Gofuku A. Applications of information and robot technologies to improve medical therapies. Siet. (2021) 20:5–9. doi: 10.1145/3427423.3427466

[24] Huang W Ai Y Chen Z Wu Q Ouyang H Jiao P. (2007). Computer supported cooperative work (CSCW) for telemedicine. 2007 11th International Conference on Computer Supported Cooperative Work in Design. 1063–1065.

[25] Jia Y Wang H Yu K Shi K Wan M. (2022). Application of computer simulation Technology in the Research of metering visual intelligent blood transfusion device. 2022 11th International Conference on Information Communication and Applications (ICICA). 18–21.

[26] Kaya İ Çolak M Terzi F. Use of MCDM techniques for energy policy and decision-making problems: a review. Int J Energy Res. (2018) 42:2344–72. doi: 10.1002/er.4016

[27] Kennedy-Metz LR Mascagni P Torralba A Dias RD Perona P Shah JA. Computer vision in the operating room: opportunities and caveats. IEEE Trans Med Robot Bionics. (2020) 3:2–10. doi: 10.1109/TMRB.2020.3040002 33644703 PMC7908934

[28] Kittipanya-ngam P Yu X Eng H-L. Computer vision Technologies for Monitoring System in tele-physiotherapy. i-CREATe '09. (2009). doi: 10.1145/1592700.1592718

[29] Lei L Li J Li W. Assessing the role of artificial intelligence in the mental healthcare of teachers and students. Soft Comput. (2023):1–11. doi: 10.1007/s00500-023-08072-5, PMID: 37362257 PMC10072038

[30] Liu N Xu Z. An overview of ARAS method: theory development, application extension, and future challenge. Int J Intell Syst. (2021) 36:3524–65. doi: 10.1002/int.22425

[31] Lundstrom A Fernaeus Y. The disappearing computer science in healthcare VR applications. Httf. (2019) 2019:3398. doi: 10.1145/3363384.3363398

[32] McKenna MK. An occupational health nursing computer application in medical care: an army approach. The seventh annual symposium on computer applications in medical care, 1983. PRO. (1983):537–9.

[33] Pourreza H Camorlinga S Leung CKS Martin BD. An information and communication technology system to support rural healthcare delivery. Ihi. (2010) 10:440–4. doi: 10.1145/1882992.1883059

[34] Toygar A Yildirim U İnegöl GM. Investigation of empty container shortage based on SWARA-ARAS methods in the COVID-19 era. Eur Transp Res Rev. (2022) 14:8. doi: 10.1186/s12544-022-00531-8 PMC8934722 38624938

[35] Whig P Velu A Nadikattu RR Alkali YJ. Computational science role in medical and healthcare-related approach. Handbook Comp Sci. (2023):245–72. doi: 10.1002/9781119763468.ch12

[36] Zhi-Gen H Jian-Ping L Lei H Yujun Y. (2015). Research and application of data warehouse and data mining technology in medical field. 2015 12th International Computer Conference on Wavelet Active Media Technology and Information Processing (ICCWAMTIP). 457–460.

[37] Zhu Y Huang R Wu Z Song S Cheng L Zhu R. Deep learning-based predictive identification of neural stem cell differentiation. Nat Commun. (2021) 12:2614. doi: 10.1038/s41467-021-22758-0, PMID: 33972525 PMC8110743

